# A Case of Recurrent Diabetic Foot Ulcers With Multi-drug Resistant Poly-Microbial Infections

**DOI:** 10.7759/cureus.65432

**Published:** 2024-07-26

**Authors:** Radha Kunjalwar, Aditya S Pedaprolu, Akshunna Keerti, Achal Chaudhari

**Affiliations:** 1 Microbiology, Jawaharlal Nehru Medical College, Datta Meghe Institute of Higher Education and Research, Wardha, IND; 2 General Surgery, Jawaharlal Nehru Medical College, Datta Meghe Institute of Higher Education and Research, Wardha, IND; 3 Internal Medicine, Jawaharlal Nehru Medical College, Datta Meghe Institute of Higher Education and Research, Wardha, IND

**Keywords:** multi-drug resistant infection, diabetes mellitus, diabetic foot ulcer, diabetic foot infections, amputation

## Abstract

For people with diabetes, diabetic foot ulcers (DFUs) are a serious condition that can result in amputations, among other dire consequences. This case report details the clinical course and management of a 40-year-old male with type II diabetes mellitus presenting with recurrent DFUs and blackening of the toes. Despite initial surgical intervention and aggressive antimicrobial therapy, the patient experienced persistent infection and graft failure, necessitating multiple treatments and ongoing care. Most of the bacteria that were identified from diabetic foot infections (DFIs) were gram-negative, and they were resistant to common treatments. The intensity of DFI was correlated with polymicrobial illnesses.

## Introduction

Around 10% of the global population suffers from diabetes mellitus (DM), and one of the most crippling and financially devastating illnesses is an unhealed diabetic foot ulcer [1]. The prevalence of diabetes mellitus is increasing worldwide and is expected to reach 366 million by the year 2030 [2]. A significant consequence of DM is foot infection, which is typically caused by polymicrobial agents. Amputation results are worse in smokers, elderly patients with a longer history of uncontrolled diabetes, and those with extensive ulcers and gangrenous infections [3]. Because there is not enough information on the environmental factors of ulcer infections and how to manage or eradicate this kind of chronic infection, ulcer infections are much too frequently followed by amputation [4]. Diabetes patients frequently experience diabetic foot ulcers, which are a major complication that frequently results in high rates of morbidity and death. Patients with diabetes mellitus have a higher morbidity and mortality rate when they have foot ulcers. Long-term hospitalization is necessary for diabetic patients with foot ulcers, as they run the danger of losing a leg. It takes effective care to avoid serious consequences like amputations [2]. This report presents the case of a patient with recurrent diabetic foot ulcers (DFUs) associated with polymicrobial infection.

## Case presentation

A 40-year-old male, a known case of type two diabetes mellitus for four years was admitted to the surgery ward with complaints of an ulcer and blackening over the fourth digit for one and a half months. He developed an ulcer over the third interdigital space post-trauma (road traffic accident) for which disarticulation of the third toe of his left foot was done. At outpatient clinics, he received wound dressings and occasionally antibiotics. The patient now came with a blackish discoloration over the fourth toe of the left toe with an ulcer over the interdigital space between the second and third toes involving the entire medial aspect of the third toe and the lateral aspect of the second toe. Routine investigations on the day of admission revealed hemoglobin (Hb) 9.5 gm/dL, white blood cell count (WBCs) 7400/mm^3^, platelets 3.35 lakh/µL, liver and kidney function tests, sodium, and potassium were within normal limits. Random blood sugar was 75 mg/dL within the normal limits. Glycated hemoglobin (HbA1C) was also maintained because of the regularity of the treatment.

**Figure 1:**
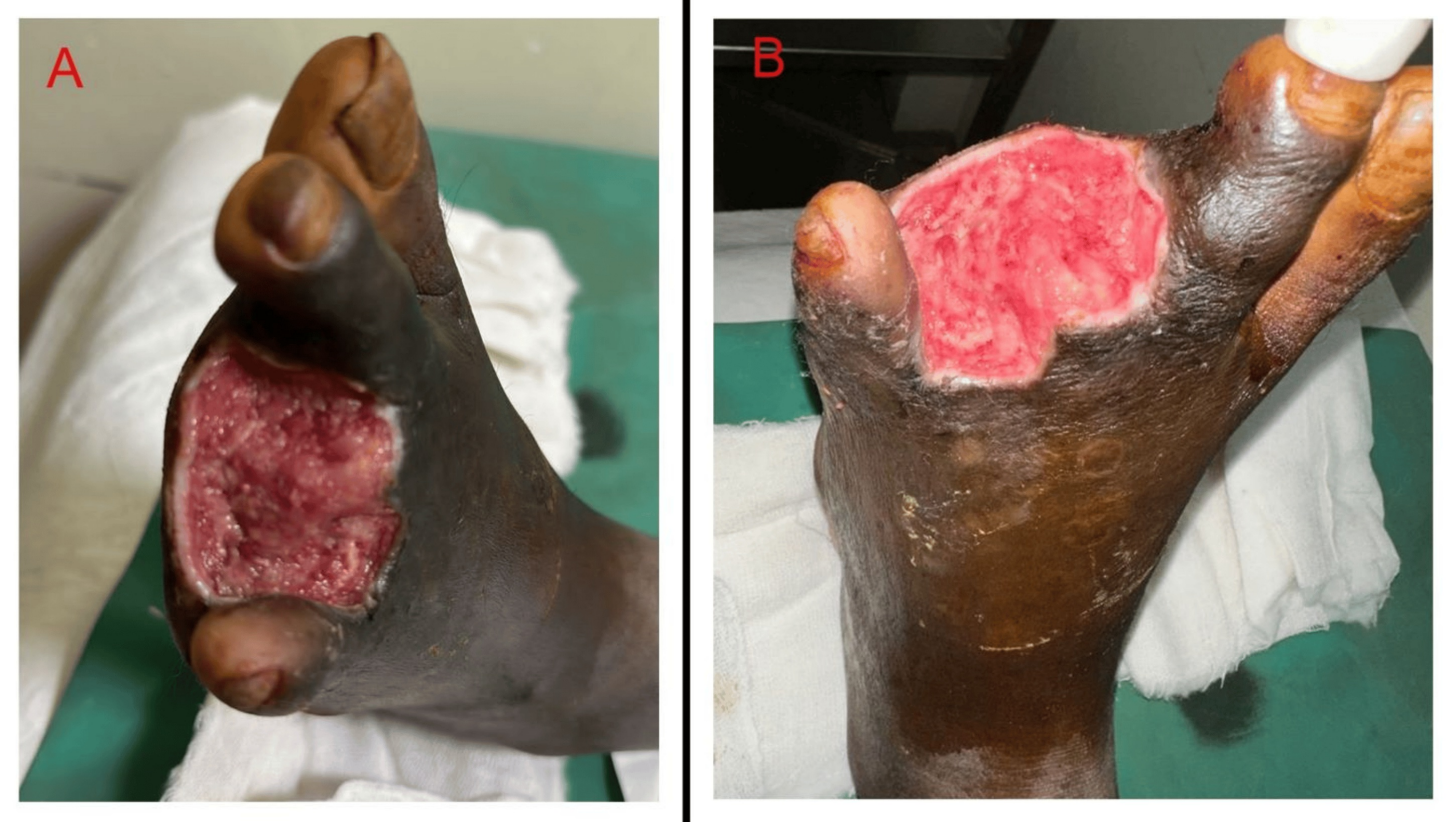
Disarticulation of the fourth toe

He was on tab glimepiride-pioglitazone and metformin for four years. Systemic examination was normal. A local examination of the ulcer revealed between the second and fourth digits and the fourth and fifth digits without pus or blood discharge. Peripheral pulsations were present but diminished, and discoloration of skin was seen. Pitting pedal edema was noted extending from foot to ankle. Blackish discoloration is present over the fourth toe of the left toe extending up to the left lower tibial region with an ulcer over the interdigital space between the second and third toes. Ultrasonography (USG) color Doppler illustrated bilateral lower limb chronic venous insufficiency. Informed consent was taken and complications including multiple surgeries and prolonged stay in hospital were explained. On day 3, the patient underwent amputation of the fourth toe shown in Figure 1, as per the surgeon's advice whose histopathological reports showed necrosis, infiltration of polymorphs, and proliferation of fibroblasts from the ulcer area. Sections from bone revealed no involvement in bony trabeculae without any establishment of gangrene.

**Figure 2:**
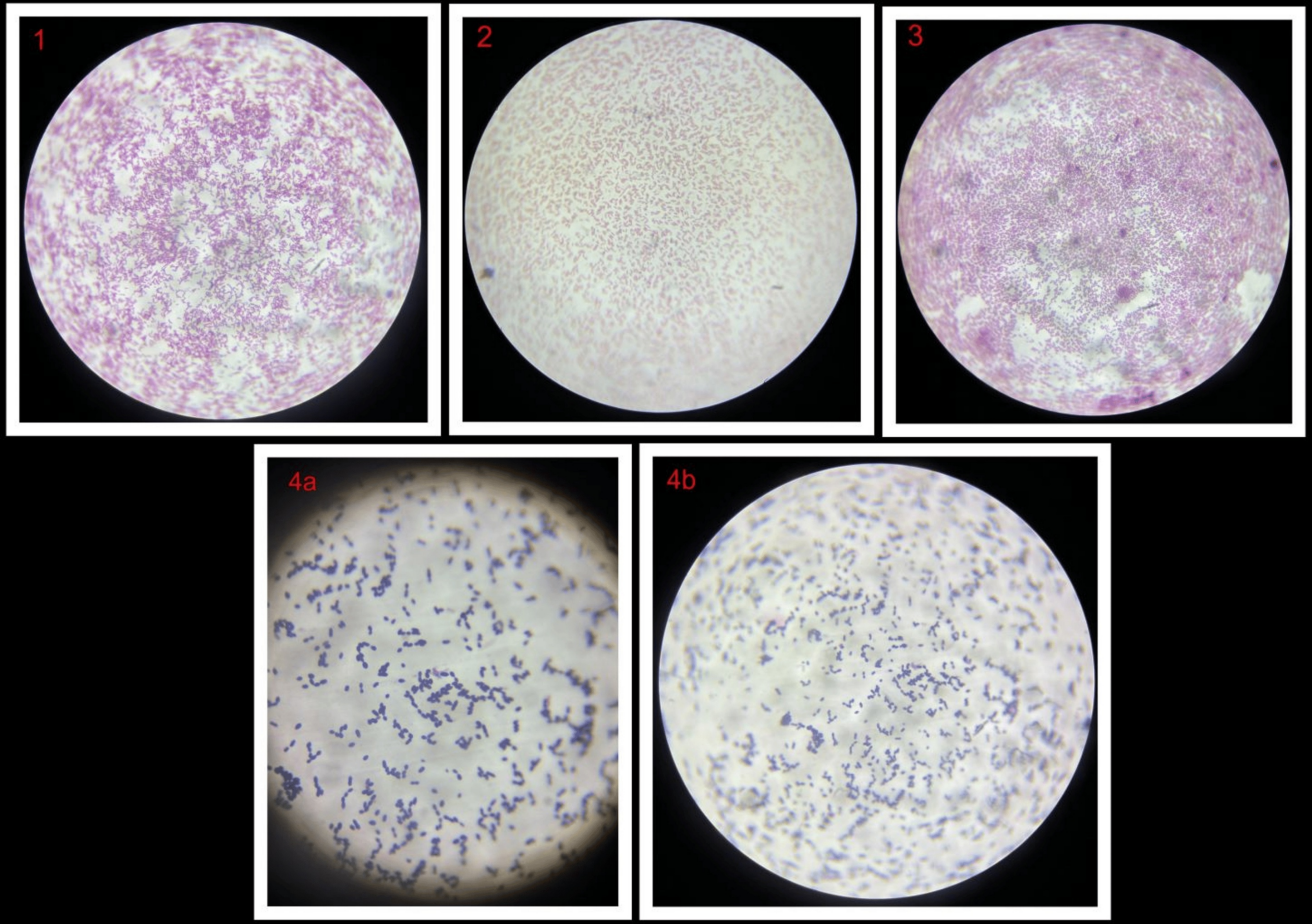
Gram staining from cultures showing (1) GNB, (2) GNB, (3) GNC, (4a&b) GPC in pairs GPC: Gram-positive cocci, GNC: Gram-negative cocci, and GNB: Gram-negative bacilli

Wound samples were received in the Microbiology laboratory on different occasions. *Klebsiella pneumoniae* was isolated on three occasions, viz., on days 6, 14, and 25 with the same resistant pattern while *Acinetobacter spp* was isolated on day 25 one time along with the *Klebsiella pneumoniae*. The identification and antimicrobial sensitivity were done by conventional Kirby-Bauer disk diffusion of these organisms, which is illustrated in Table 1. On day 31, again the wound sample was sent to the laboratory, and the culture was found positive for Enterococcus faecalis. This pathogen was sensitive to ampicillin, vancomycin, linezolid, and high-level gentamycin (HLG), and resistant to azithromycin, penicillin, and ciprofloxacin. Gram staining from the culture is shown in Figure 2.

**Table 1:** Antimicrobial sensitivity testing by the conventional Kirby-Bauer disk diffusion method R: Resistant, S: Sensitive

Antibiotics	Klebsiella pneumoniae	Acinetobacter spp.
Ampicillin	R	R
Amoxiclav	R	R
Cotrimoxazole	R	R
Ciprofloxacin	R	R
Ceftriaxone	R	R
Cefotaxime	R	R
Cefepime	R	R
Ceftazidime	R	R
Cefuroxime	R	R
Aztreonam	R	R
Amikacin	R	R
Gentamycin	S	S
Piperacillin/tazobactam	R	S
Imipenem	R	R
Meropenem	R	R
Tigecycline	S	S
Colistin	S	S

Initially, he was managed with surgical debridement and empirical antibiotic treatment with intravenous amoxiclav 625 mg for 7 days and tab linezolid 600 mg for 5 days before the sensitivity report. After sensitivity report gentamicin 80 mg for 12 days was given. He underwent a split skin graft over the left third and fourth disarticulated digital space. Slough was seen over the lateral aspect of the graft site on day 9 (postoperative day 6), the graft was infected, and hence sloughed-out graft was removed and treated with local mupirocin. He was discharged in a stable condition after being revived for blood sugar levels. Initial graft placement was uneventful; however, infection and graft failure were noted by day 9 (postoperative day 6). The sloughed-out graft was removed, and local mupirocin cream was applied.

Ongoing care by daily sterile dressings and continued monitoring of blood glucose levels was done. The patient was discharged in stable condition with the following recommendations for continued oral hypoglycemic agents (empagliflozin-linagliptin, glimepiride), regular follow-up in the outpatient department, and daily sterile dressings for the ulcer site.

## Discussion

Diabetic foot ulcers (DFUs) will strike 13% of diabetes patients at least once, and 59% of DFU patients will experience a recurrence over the next five years [1,2,5]. DFUs are complex to manage due to the interplay of vascular insufficiency, infection, and poor glycemic control. A percentage of diabetes individuals are admitted to hospitals each year due to foot infections [6]. Diabetes increases the risk of bacterial infections spreading quickly from diabetic foot ulcers, resulting in permanent tissue damage [7]. Despite optimal surgical and medical interventions, this patient faced significant healing challenges resulting in recurrent non-healing ulcers, highlighting the need for comprehensive and multidisciplinary care approaches in managing diabetic foot complications. Differential sensitivity patterns were seen in isolated bacteria when exposed to widely used antibiotics. Most of the isolates exhibited resistance to multiple antibiotics that are typically administered based on empirical evidence [6]. Factors always have had a role in the development of chronic DFU: malformation, deep infection, and hypoxemia. This troublesome trio is an indirect offender along with neuropathy [1]. Luckily in our case, though the patient had trauma that led to the ulcer formation, the severity of these symptoms is not established. We observed the established polymicrobial infection predominantly. We found similar findings in another Indian study [6]. The literature has extensively documented the polymicrobial nature of DFIs [3,8]. According to our investigation, mild DFI can be mostly brought about by monomicrobial infections but moderate to severe DFI involves infections by polymicrobial species.

In our scenario, the gram-negative organisms were multi-drug resistant (MDR) in both instances. The organisms were sensitive to only gentamycin, colistin, and tigecycline. The piperacillin/tazobactam was sensitive in the case of Acinetobacter spp only, which was similar to one study [6]. Gentamycin was sensitive on all occasions, which could be begun empirically based on the clinical signs of the illness and adjusted after knowing the outcome. On the other hand, the organism was sensitive to both colistin and tigecycline, so they can be kept as a last resort for gram-negative organisms. Similar findings were seen in another study [3]. Carbapenem resistance is a global problem and becoming very difficult to treat day by day. We also report the carbapenem resistance in our case. For the single gram-positive bacteria, ampicillin, vancomycin, and linezolid were found to be the most effective drugs overall. This finding was in concordance with other pieces of literature [3,9,10]. Foot ulcers are a preventable ailment for which modest interventions through programs that could lower their risk factors can reduce amputations by as much as 70% [5]. Our patient had some of the risk factors for developing the DFUs such as male gender, history of DM, age, history of trauma, and smoking. DFUs have a substantial impact on a person's social and mental well-being because the likelihood of amputation is considerable and can significantly damage a patient's quality of life [2]. So the use of sterile gloves and maintaining the proper sterile environment in the hospital can limit the spread of infection. Gram-negative bacteria were isolated, and they continued to be resistant to commonly used antibiotics on different occasions. Despite proper and regular treatment for diabetes mellitus, the patient developed ulcers, which led to amputation.

## Conclusions

The majority of the pathogens found in diabetic foot infections were gram-negative and only a single incidence of gram-positive organism was noted. This case underscores the difficulties in treating diabetic foot ulcers, particularly in the presence of venous insufficiency and recurrent infections. The presence of venous insufficiency and uncontrolled blood sugar levels are considered to be significant risk factors for the development of recurrent diabetic foot ulcers. Also, a combination of antimicrobial agents should be used for treating polymicrobial infections depending on the antimicrobial susceptibility pattern. The majority of the resources should be focused on emphasizing the control of blood sugar levels, foot care practices, regular monitoring of blood sugar levels, and timely follow-ups as suggested by healthcare professionals.
